# Thyroid Function of Pregnant Women and Perinatal Outcomes in North Macedonia

**DOI:** 10.1055/s-0041-1736172

**Published:** 2021-11-16

**Authors:** Maja Avramovska, Neda Milevska Kostova, Borislav Karanfilski, Sandra Hunziker, Olivija Vaskova, Goran Dimitrov, Elena Dzikova, Ana Daneva Markova, Marija Hadzi Lega, Gligor Tofoski, Aleksandar Sikole

**Affiliations:** 1 Department of Obstetrics and Gynecology, Clinical Hospital “Dr. Trifun Panovski” - Bitola, Partizanska nn, North Macedonia; 2 Departement Gesundheitswissenschaften und Technologie, Institute for Social Innovation, Skopje, Skopje, North Macedonia; 3 National Committee for Iodine Deficiency, Ministry of Health, Skopje, North Macedonia; 4 Laboratory of Human Nutrition, Institute of Food, Nutrition and Health, ETH Zurich, Zurich, Switzerland; 5 Institute of Pathophysiology and Nuclear Medicine, Faculty of Medicine, Ss. Cyril and Methodius University Skopje, Skopje, North Macedonia; 6 University Clinic of Gynecology and Obstetrics, Faculty of Medicine, Ss. Cyril and Methodius University Skopje, Skopje, North Macedonia; 7 Department for Fetal Screening and Perinatal Medicine, Danat al Emarat Hospital for Women and Children, Abu Dhabi, Emirate of Abu Dhabi; 8 Department of Nephrology, University Clinic of Nephrology, Faculty of Medicine, Ss. Cyril and Methodius University Skopje, Skopje, North Macedonia

**Keywords:** thyroid stimulating hormone, total thyroxine, thyroglobulin, perinatal outcomes, low birth weight, hormônio estimulante da tireoide, tiroxina total, tireoglobulina, resultados perinatais, baixo peso de nascimento

## Abstract

**Objective** Thyroid diseases are the second most common endocrine disorders in the reproductive period of women. They can be associated with intrauterine growth restriction (IUGR), preterm delivery, low Apgar score, low birthweight (LBW) or fetal death. The aim of the present study is to explore thyroid dysfunction and its relationship with some poor perinatal outcomes (Apgar Score, low birthweight, and preterm delivery).

**Methods** Dried blood spot samples from 358 healthy pregnant women were analyzed for thyroid stimulating hormone (TSH), total thyroxine (TT4), and thyroglobulin (Tg). Neonatal data were collected upon delivery. Four groups were formed based on thyroid function tests (TFTs).

**Results** Of the 358 tested women, 218 (60.72%) were euthyroid. Isolated hypothyroxinemia was present in 132 women (36.76%), subclinical hyperthyroidism in 7 women (1.94%), and overt hypothyroidism in 1 (0.28%). The perinatal outcomes IUGR ( *p* = 0.028) and Apgar score 1 minute ( *p* = 0.015) were significantly different between thyroid function test [TFT]-distinct groups. In the multiple regression analysis, TT4 showed a statistically significant inverse predictive impact on LBW ( *p* < 0.0001), but a positive impact of Tg on LBW ( *p* = 0.0351).

**Conclusion** Thyroid hormones alone do not have a direct impact on neonatal outcomes, but the percentage of their participation in the total process cannot be neglected. Based on the regression analysis, we can conclude that TT4 and Tg can be used as predictors of neonatal outcome, expressed through birthweight and Apgar score. The present study aims to contribute to determine whether a test for thyroid status should become routine screening during pregnancy.

## Resumo

**Objetivo** As doenças da tireoide são as segundas doenças endócrinas mais comuns no período reprodutivo das mulheres. Elas podem estar associadas à restrição de crescimento intrauterino (RCIU), parto prematuro, baixo índice de Apgar, baixo peso ao nascer (BPN) ou morte fetal. O objetivo do presente estudo é explorar a disfunção tireoidiana e sua relação com alguns resultados perinatais insatisfatórios (índice de Apgar, baixo peso ao nascer e parto prematuro).

**Métodos** Amostras secas de sangue em 358 gestantes saudáveis foram analisadas para hormônio estimulador da tireoide (TSH), tiroxina total (TT4) e tireoglobulina (Tg). Os dados neonatais foram coletados no momento do parto. Quatro grupos foram formados com base em testes de função tireoidiana (TFT).

**Resultados** Das 358 mulheres testadas, 218 (60,72%) eram eutireoidianas. Hipotiroxinemia isolada estava presente em 132 mulheres (36,76%), hipertireoidismo subclínico em 7 mulheres (1,94%) e hipotireoidismo evidente em 1 (0,28%). Os resultados perinatais RCIU ( *p* = 0,028) e índice de Apgar de 1 minuto ( *p* = 0,015) foram significativamente diferentes entre os grupos distintos de TFT. Na análise de regressão múltipla, TT4 mostrou impacto preditivo inverso estatisticamente significativo no BPN ( *p* < 0,0001), mas impacto positivo da Tg no BPN ( *p* = 0,0351).

**Conclusão** Isoladamente, os hormônios tireoidianos não têm impacto direto no desfecho neonatal, mas o percentual de sua participação no processo total não pode ser desprezado. Com base na análise de regressão, podemos concluir que TT4 e Tg podem ser usados como preditores do resultado neonatal, expressos por meio do peso ao nascer e do índice de Apgar. O presente estudo tem como objetivo contribuir para que um teste para verificar o estado da tireoide deva se tornar um rastreamento de rotina durante a gravidez.

## Introduction

Thyroid diseases are the second most common endocrine disorders affecting women in the reproductive period. 1 Early diagnosis and treatment of thyroid diseases before and during pregnancy is important for maintaining the health of the mother and the baby. 2 The maternal thyroid gland undergoes physiological changes during pregnancy, including increased thyroid gland vascularity, increased iodine clearance and fluctuation in thyroxine metabolism, which may impair the maternal-fetal transfer of thyroxine despite optimal thyroid status. 3

Hypothyroidism is relatively uncommon in pregnancy, occurring in between 2 and 3% of pregnant women, mainly as subclinical form. 1 Hyperthyroidism in pregnancy, also with very low prevalence ranging between 0.1% and 1%, 4 has been associated with intrauterine growth restriction (IUGR) and low birthweight (LBW). 5 The study of the Consortium on Safe Labor found that neonates of women with hyperthyroidism were more likely to need resuscitation in the delivery room and neonatal intensive care unit (NICU) admission. Also, they had an 1.6- to 2.0-fold odds of respiratory distress syndrome, transient tachypnea, apnea, sepsis, as well as increased odds of cardiomyopathy, retinopathy of prematurity, and neonatal thyroid disease. 5

Isolated maternal hypothyroxinemia has been reported in ∼ 1.3% of pregnant women; however, its incidence can be as high as 25.4%. 6 An American study of 17,298 women examined the association between maternal isolated hypothyroxinemia and adverse neonatal outcomes, including LBW, Apgar score < 3, and fetal or neonatal mortality. 7 The same study suggested that preterm delivery may be associated with maternal hypothyroxinemia, characterizing the condition as detrimental during pregnancy. 7

According to the guideline of the American Thyroid Association (ATA) for the diagnosis and management of thyroid disease during pregnancy and the postpartum period, 8 9 the reference values for thyroid stimulating hormone (TSH) and total thyroxine (TT4) range from 0.1 to 3.7 mIU/L and 65/97.5–165–247.5 nmol/L, respectively. Based on the above, the study population was categorized into four groups.

The largest decrease in TSH is observed during the 1 ^st^ trimester because of elevated levels of serum hCG directly stimulating the TSH receptor, therefore increasing thyroid hormone production. 8 Consequently, serum TSH and its reference range gradually rise in the 2 ^nd^ and 3 ^rd^ trimesters but are still lower than in nonpregnant women.

The aim of our study is 2-fold: first, to examine the correlation between thyroid function tests (TFTs) and neonatal outcome; and second, to evaluate the predictive potential of TSH, TT4 and Tg on delivery and some of the perinatal outcomes (Apgar Score and low birth weight).

## Methods

The present prospective study included 358 healthy pregnant women without known thyroid disorders. For recruitment, the following inclusion criteria were adopted: singleton pregnancy at any gestational age, with the following exclusion criteria: no previous history of thyroid disease or treatment with any thyroid-related therapy, cigarette smoking, and no history of other chronic diseases, in particular diabetes mellitus or hypertension, and any fetal anomaly diagnosed with amniocentesis or ultrasound. Pregnant women were divided into 3 subgroups depending on the gestational age at the time of recruitment: 1 ^st^ trimester (up to the 12 ^th^ gestation week [g.w.], second trimester (between the 12 ^th^ and 28 ^th^ g.w.), and 3 ^rd^ trimester (29 g.w. to the end of pregnancy).

Participant recruitment and collection of blood samples were performed between April and July 2017. Data on maternal age, weight and height, gestational age at the time of recruitment, as well as eligibility check against exclusion criteria, were collected using a recruitment questionnaire.

Dried blood spot (DBS) samples were collected upon recruitment onto filter paper cards using the standard lancet finger prick method. The samples were dried for 24 hours and stored at - 20°C. The TSH and TT4 levels were analyzed with a time-resolved fluoroimmunoassay method (GSP 2021–0010; PerkinElmer, Turku, Finland) at the University Children's Hospital, Zurich, Switzerland. The Tg concentration was assessed using a DBS Tg sandwich enzyme-linked immunosorbent assay 10 at Eidgenössische Technische Hochschule Zürich (ETH Zurich).

Postpartum data was retrieved for each of the labored women from their medical histories. Birthweight and birth length were measured by the midwife attending the birth, while condition of the newborn after delivery and Apgar scores were determined by the neonatologist. In addition, obstetric history, gestational age at the time of birth, as well as the way of birth were noted in the medical history.

Intrauterine growth restriction (IUGR) was defined as birthweight < 10 ^th^ percentile for the gestational age. Low birthweight (LBW) was defined as weight ≤ 2,500 g, regardless of the gestational age. 11 Preterm delivery was defined as delivery before 37 completed g.ws.. Low Apgar score was considered if Apgar score at 1 minute was < 7. 12

All participants signed an informed consent form after reading the project information sheet, and the ethics approval for the study was obtained from the Etic Committee at Medical Faculty – Skopje, Ss. Cyril and Methodius University – Skopje at its XVI session on January 24, 2019, with N * 03–242/3.

Data analyses were performed using MedCalc Statistical Software version 19.1.3 (MedCalc Software, Ostend, Belgium). Continuous variables were presented as mean ± standard deviation (SD), median (interquartile range [IQR]) (when the frequency distribution for our data was skewed) and percentages (%). One-way analysis of variance (ANOVA) was used to test the difference between the means of the six subgroups. Prior to the ANOVA test, the Levene test for equality of variances was performed. We applied a logarithmic transformation if the Levene test was positive ( *p* < 0.05). Multiple backward regression analysis was used to show predictable values of independent variables (TT4 and Tg as predictors) on the dependent variable (LBW).

## Results

In total, 358 women were included in the analysis. The mean maternal age was 29.27 ± 5.5 years old (range: 25 to 33 years old), their BMI was overweight, preobesity state according to the World Health Organization (WHO) classification 13 (27.12 ± 4.4 kg/m ^2^ ; range: 23.44–29.63 kg/m ^2^ ), delivered approximately at 38.43 ± 2.54 g.ws. Of the total sample, 64 women were classified at the 1 ^st^ trimester according to g.w. (17.82%), 100 were in the 2 ^nd^ (27.85%), and 194 were in the 3 ^rd^ trimester (54.03%) ( Table 1 ). The median (IQR) TSH of the women in the 1 ^st^ trimester did not deviate from the reference range (0.4 mIU/mL; range 0.3 mIU/mL), whereas the TT4 values of only 2 pregnant women (3.12%) deviated from it. In the 2 ^nd^ trimester *,* from all the cohort, the median TSH was within the reference values (0.4 mIU/mL; range 1.1 mIU/L), while the TT4 values of 31 pregnant women (31%) deviated from the reference range. In the 3 ^rd^ trimester, the median TSH of 0.56 mIU/L; (range 0.4 mIU/L) did not deviate from reference range; however, the TT4 values of 54 (27.83%) pregnant women deviated from the reference range. Table 1 summarizes the clinical and demographic characteristics of the study population at the time of recruitment.

**Table 1:** Demographic and clinical characteristics of the study population at recruitment

Demographic values	*n*	% of total
1 ^st^ trimester	64	17.82%
2 ^nd^ trimester	100	27.85%
3 ^rd^ trimester	194	54.03%
	Mean ± SD	25 ^th^ – 75 ^th^ P (min - max)
Age (years old)	29.27 ± 5.5	25 - 33
BMI (kg/m ^2^ )	27.14 ± 4.7	23.44–29.63
Gestational week at birth	38.43 ± 2.54	37.7–40.0
Thyroid function values		
TSH (mU/L)	0.54 ± 0.33	0.3–0.7
TT4 (nmol/L)	103.92 ± 28.7	24 - 195.2
Tg (µg/L)	11.6 ± 9.01	5.53–15.49

Abbreviations: BMI, body mass index; SD, standard deviation; Tg, thyroglobulin; TSH, thyroid stimulating hormone; TT4, total thyroxine.

The results are expressed as median (interquartile range) or mean ± SD.

Of the cohort of 358 tested women, 193 pregnancies were terminated with normal vaginal delivery (53.91%), while 165 women underwent cesarean section (46.08%). From all the births, 41 delivered prematurely (before the 37 ^th^ g.w.) corresponding to 11.45%, and only 21 infants had an Apgar score value at 1 minute < 7 (5.86%). Low birthweight was noted in 47 cases (13.12%). When we categorized the pregnant women according to their gestational age and TFT, we noticed that, from the total of the group, 218 mothers (60.72%) were euthyroid. Isolated hypothyroxinemia was present in 132 women (36.76%), subclinical hyperthyroidism in 7 women (1.94%), overt hypothyroidism in 1 (0.28%), and no cases (0%) with subclinical hypothyroidism or overt hyperthyroidism ( Table 2 ).

**Table 2:** Categorization of pregnant women based on gestational age and TFT

Group	TSH level	TT4 level	No. (%) of pregnant women in the group
1. Normal thyroid function	normal **(** 0.1 to 3.7 mIU/L)	normal, slightly increased	218 (60.72%)
2. Isolated hypo thyroxinemia	normal **(** 0.1 to 3.7 mIU/L)	depressed	132 (36.76%)
3. Subclinical hyperthyroidism	suppressed (<0.1 mIU/L)	normal	7 (1.94%)
4. Subclinical hypothyroidism	elevated (3 to 10 mIU/L)	normal	0 (0.0%)
5. Overt hypothyroidism	elevated (> 3.7 mIU/L)	low (for TSH < 10mIU/L) any level (for TSH ≥ 10 mIU/L	1 (0.27%)
6. Overt hyperthyroidism	suppressed (< 0.1 mIU/L)	high (or irrelevant)	0 (0.0%)

Abbreviation: TSH, thyroid stimulating hormone.

Table 3 shows the way of birth in each group. A total of 58.25% of the pregnant women with normal thyroid function, 46.21% of the isolated hypothyroxinemia group, and 71.42% of the subclinical hyperthyroidism group had normal spontaneous delivery. Also, the fetal outcome in each group is shown in Table 3 . Adverse fetal outcome in isolated hypothyroxinemia included preterm delivery (9.84 versus 0 versus 12.84%), IUGR (2.27 versus 0 versus 4.12%), and LBW (12.87 versus 14.28 versus 13.30%) as compared with the group of subclinical hyperthyroidism and normal thyroid function group, consequently. Apgar score (1 minute) < 7 was seen in the group of subclinical hyperthyroidism in 28.57% of the cases, compared with the group of normal thyroid function, which appears with 5.96%. and the isolated hypothyroxinemia group, with 4.54%. The median BMI was 26.44 kg/m ^2^ for women with normal thyroid function, 27.96 ± 5.05 kg/m ^2^ for women with isolated hypothyroxinemia, and 23.23 kg/m ^2^ for those with subclinical hyperthyroidism ( *p* = 0.018). Obese women had depressed TT4 range and were prone to hypothyroxinemia. Substantial differences between the four groups in accordance with their impact on the neonatal outcome are presented by ANOVA results: F-ratio (F) and p-value of significance ( *p* ) are shown in Table 3 . The statistical significance of each group differs according to certain variables shown: Apgar score (1 minute) (F = 4.252; *p* = 0.015), IUGR (F = 3.594; *p* = 0.028), and mean BMI (F = 4.091; *p* = 0.018). A greater F-ratio value and A smaller *p* mean a higher difference between the groups according to outcomes, according to thyroid status that defines groups.

**Table 3:** Mode of delivery and fetal outcomes in different groups

Groups	1	2	3	4		
Mode of delivery	Normal thyroid function ( *n* = 218)	Isolated hypothyroxinemia ( *n* = 132)	Subclinical hyperthyroidism ( *n* = 7)	Overt hypothyroidism ( *n* = 1)	F	*p-value*
Normal spontaneous delivery	127 (58.25)	61 (46.21)	5 (71.42)	0	2.6975	0.068
CS total	91 (41.74)	71 (53.78)	2 (28.57)	1		
- CS for fetal distress	28 (30.76)	10 (7.57)	1 (14.28)	1	1.1334	0.323
- CS for dystotia/dysproprtion	25 (27.47)	11(8.33)	1 (14.28)	0	4.175	0.024
Fetal outcomes						
Preterm births	28 (12.84)	13 (9.84)	0	0	0.804	0.448
IUGR	9 (4.12)	3 (2.27)	0	0	3.594	0.028
LBW	29 (13.30)	17 (12.87)	1 (14.28)	0	0.517	0.597
Apgar score (1 minute) < 7	13 (5.96)	6 (4.54)	2 (28.57)	/	4.252	0.018
Mother characteristics						
Median age (years old)	29	29	32	26	1.299	0.274
Median BMI, kg/m ^2^	26.44	27.43	23.23	27.12	4.091	0.018

Abbreviations: BMI, body mass index; CS, cesarean section; IUGR, intrauterine growth restriction; LBW, low birthweight.

The results of the Levene test show significance only for Apgar score (1 minute). Levene statistic (LS = 7.897) and p-value ( *p* < 0.001). The Levene test for IUGR (LS = 1.643; *p* = 0.179) and BMI (LS = 1.3; *p* = 0.274) do not show statistical significance. Assessments (standardized coefficient β [βst], standard error of βst [Std. Error], t, p-value, partial r [r _partial_ ], semipartial r [r _semipartial_ ], and exp [βst]) of the dependent predictor LBW or determinants (TT4 and Tg) for increasing the incidence of LBW in TFT-categorized groups after backward multiple regression analysis are shown in Table 4 .

**Table 4:** Multiple backward regression analysis of low birthweight according to thyroid status categorization groups

**Dependent Y**	**LBW**						
**Regression Equation**							
**Independent variables**	**βst coefficient**	**Std. Error**	**t**	***p-value***	** r _partial_**	** r _semipartial_**	**exp (βst)**
(Constant)	2.5225						
TT4	- 0.01124	0.0009484	- 11.854	< 0.0001	- 0.5391	0.5388	0.98767
Tg	0.006294	0.002975	2.116	0.0351	0.1135	0.09616	1.00631
Significance level							***p*** ** < 0.0001**

Abbreviations: βst, β standardized; LBW, low birthweight; Std, standard; Tg, thyroglobulin; TT4 = total thyroxine.

Groups 3 and 4 were excluded from the regression analysis due to small group size. The p-values followed the order of statistical significance: TT4 (< 0.0001) and Tg (0.0351). Due to the regression model criterion (remove variable if *p* > 0.5), TSH was not included in the regression analysis. There was an inverse correlation (negative βst coefficient; βst = - 0.01124) between TT4 and LBW, and a positive correlation (positive βst = 0.006294) between Tg and LBW. The regression parameter exp (βst) for TT4 (1.012484 = 1/0.98767) signified that with each increase of 1 unit (nmol/L) in TT4, the LBW score decreased by 1.01248 g. The regression parameter exp (βst) for Tg (1.00631) signified that with each increase of 1 unit (μg/L) in Tg, the LBW score increased by 1.00631 g. The coefficient of determination R ^2^ (0.2914) and the multiple correlation coefficients (0.5398) showed that 29.14% from LBW was dependent on TT4 and Tg as the predictors. Only 29.14% of the changes in LBW were a result of TT4 changes (accompanied by Tg changes), and the remaining from the total variability between them were not explained (70.86% of LBW were dependent on other factors, which were not covered with the regression model. Partial coefficient of regression βst for Tg affects the LBW, because there is a simple linear relationship between Tg and LBW and it can be used for predicting. In addition, TT4 is in a simple linear relationship with LBW, and it can also be used for predicting LBW.

## Discussion

Attention to thyroid dysfunction during pregnancy has increased in the past decade, particularly in the area of subclinical thyroid dysfunction, as thyroid disease is the second most common endocrine disorder complicating pregnancy. 14 We examined the thyroid function through TSH and TT4 and its relationship with perinatal outcomes in 358 healthy pregnant women divided in subgroups created by ATA guidelines.

We revealed that thyroid dysfunction during pregnancy was associated with preterm delivery, low Apgar score and low birth weight (< 2,500 g), even in the forms of isolated hypothyroxinemia. The study of Saki et al. 15 showed that both hyperthyroidism and hypothyroidism are associated with IUGR, contrary to our results, in which isolated hypothyroxinemia was connected with IUGR. They found that hypothyroidism was associated with IUGR ( *p* = 0.017) and low Apgar score in the first minute ( *p* = 0.04); thus, the risk for IUGR was increased by 2.2 times, and the low Apgar score increased the risk by 1.95 times. Intergroup comparison in our study showed statistically significant differences with respect of IUGR ( *p* = 0.028) and Apgar score [(1 minute) < 7] ( *p* = 0.018), results similar and close to the above-mentioned studies.

Clinical hypothyroidism also showed a statistically significant correlation with premature labor ( *p* = 0.045) in the study by Saki et al. 15 According to Davis et al., 16 premature labor occurred in 44% of pregnant women diagnosed with hypothyroidism and in 17% of pregnant women with subclinical hypothyroidism, which is similar to our results for preterm births (18% of women with subclinical hypothyroidism labored prematurely).

Millar et al. 17 also reviewed pregnancy outcomes in 181 women with hyperthyroidism and demonstrated that uncontrolled hyperthyroidism was associated with a 9-fold higher LBW rate compared with the control population, which is not different from our study results. We found that TT4 ( *p* < 0.0001) and Tg ( *p =* 0.0351) have a simple linear relationship with LBW and can be used for predicting it. Based on the multiple correlation coefficient in our study, it is calculated that 53.98% from LBW was dependent on TT4 and Tg as the predictors.

In our study, isolated hypothyroxinemia was associated with increased risk of preterm delivery (3.62% of all pregnancies) and with increased rate of cesarean section delivery (51.58%). Also, LBW (< 2,500 g) was found in 11.90% of the newborns and lower Apgar score (1 minute) < 7 (2.38%). Subclinical hyperthyroidism had association with increased risk of preterm delivery (18%), as well as low Apgar score at the 1 ^st^ minute (25%) and LBW < 2,500 g (25%).

According to Davis et al., 16 perinatal mortality and morbidity were also increased due to placental abruption (19%), as well as to postpartum hemorrhage and anemia (19%), with consequent LBW (31%) or even fetal death (12%). Leung at al. 18 found that the pregnancies with overt hypothyroidism had significant increase in the incidence of LBW of the neonates (< 2,500 g) compared with controls. Others found no association between thyroid hormonal status or thyroid antibody positivity and preterm delivery or other obstetrical complications, like in the study of Lejeune et al. 19 In a study of 233 pregnant women with isolated hypothyroxinemia, Casey et al. 20 reported no increased adverse perinatal outcomes associated with the condition.

The present study has limitations. First, although the results of the study were statistically significant for some of the analyses, the number of women with thyroid dysfunction was limited, especially in groups with subclinical hyperthyroidism and overt hypothyroidism. Second, neither thyroid peroxidase antibodies (TPOAb), nor thyroglobulin antibodies (TgAb) were evaluated by us, which are also connected to negative neonatal outcomes. In the future, a bigger cohort plus TPOAb and TgAb evaluation is a logical next step.

## Conclusion

Thyroid hormones alone do not have a direct impact on neonatal outcome. However, even if it was small, the percentage of their participation in the total process that affect the final outcome cannot be neglected. Based on the regression analysis, we can conclude that TT4 and Tg can be used as determinants for predicting the neonatal outcome, expressed through birthweight and Apgar score. These results are similar to the data that had been evaluated over the past 10 years, although there are still ongoing studies trying to clarify whether maternal subclinical hyperthyroidism and isolated hypothyroxinemia in pregnancy are associated with adverse outcomes. The present study aims to contribute to the scientific debate as to whether a test for thyroid status should become routine screening during pregnancy.
